# The caspase-inhibitor Emricasan efficiently counteracts cisplatin- and neomycin-induced cytotoxicity in cochlear cells

**DOI:** 10.1007/s00109-024-02472-2

**Published:** 2024-08-07

**Authors:** Larissa Nassauer, Juliane W. Schott, Jennifer Harre, Athanasia Warnecke, Michael Morgan, Melanie Galla, Axel Schambach

**Affiliations:** 1 Institute of Experimental Hematology, Hannover Medical School, Carl-Neuberg-Strasse 1, D-30625 Hannover, Germany; 2 Department of Otorhinolaryngology, Head and Neck Surgery, Hannover Medical School, 30625 Hannover, Germany; 3 Cluster of Excellence “Hearing4all”, Hannover Medical School, 30625 Hannover, Germany; 4 Division of Hematology/Oncology, Boston Children’s Hospital, Harvard Medical School, Boston, MA 02115 USA

**Keywords:** Hearing loss, Ototoxicity, Cisplatin, Neomycin, Anti-apoptotic, Caspase inhibitor

## Abstract

**Abstract:**

Cisplatin is a chemotherapeutic agent widely used to treat solid tumors. However, it can also be highly ototoxic, resulting in high-frequency hearing loss. Cisplatin causes degeneration of hair cells (HCs) and spiral ganglion neurons (SGNs) in the inner ear, which are essential components of the hearing process and cannot be regenerated in mammals. As the affected cells primarily die by apoptosis, we tested several anti-apoptotic small molecules to protect these cells from drug-induced toxicity. We found that the general caspase inhibitor Emricasan could significantly counteract the toxic effects of cisplatin in House Ear Institute-Organ of Corti 1 (HEI-OC1) cells, phoenix auditory cells, and primary SGNs. Importantly, the anti-cytotoxic effect in neuronal cells was even more pronounced than the effect of sodium thiosulfate (STS), which is currently the only approved prevention option for cisplatin-induced ototoxicity. Finally, we tested the protective effect of Emricasan treatment in the context of another ototoxic drug, i.e., the aminoglycoside antibiotic neomycin, and again found a significant increase in cell viability when the cultures were co-treated with Emricasan. These results suggest a promising strategy to prevent ototoxicity in patients by temporarily blocking the apoptotic pathway when applying cisplatin or aminoglycoside antibiotics.

**Key messages:**

- Anti-apoptotic small molecules can reduce cisplatin-induced toxicity.
- Emricasan can effectively exert its anti-apoptotic effect on cochlear cells.
- Strong protection from cisplatin- and neomycin-induced cytotoxicity with Emricasan.
- Sodium thiosulfate and Emricasan provide similar protective effects to cisplatin-treated cells.
- Emricasan is more potent than sodium thiosulfate in reducing neomycin-induced cytotoxicity.

**Supplementary information:**

The online version contains supplementary material available at 10.1007/s00109-024-02472-2.

## Introduction

Over 200 drugs are associated with ototoxicity, an adverse reaction that affects the inner ear or auditory nerve, leading to cochlear or vestibular dysfunction. Examples of ototoxic drugs include aminoglycoside antibiotics and platinum-based chemotherapeutic agents [1]. The incidence of ototoxicity ranges from 7–90% and 50–90%, depending on several factors that influence the severity, such as the type of drug administered [2, 3]. Among the aminoglycosides, neomycin is considered the most ototoxic, while cisplatin is highly ototoxic among platinum-based derivatives [4]. Although both groups of drugs have been known to exhibit ototoxicity since the 1970s, they remain in use due to their high efficiency in treating bacterial infections or tumors, respectively [5, 6]. Patients who experience ototoxicity suffer from permanent, bilateral, and progressive tinnitus and report high-frequency hearing loss. The use of aminoglycosides also leads to vestibular toxicity, with symptoms such as dizziness and vertigo [7, 8]. Treatment with ototoxic drugs primarily leads to dysfunction and degeneration of HCs and SGNs, which are the core components of the auditory system and are both incapable of regeneration in mammals [9–12]. Previous studies have shown that the ototoxic effect is closely related to the accumulation of reactive oxygen species (ROS) and the apoptosis of affected cells [13–15].

Several strategies have been tested for protection from ototoxicity. These mainly focused on small molecules due to their numerous advantages: they are easy to synthesize, have a long shelf life, and can be easily distributed and penetrate cell membranes to reach intracellular compartments [16]. Studies using small molecules have aimed to support the antioxidant defense system by increasing ROS scavenging or preventing apoptosis in affected cell types. STS, N-acetylcysteine, and dexamethasone were shown to be efficient ROS scavengers, whereas the caspase-9 or caspase-3 inhibitors Z-LEHD-FMK and Z-DEVD-FMK, respectively, prevented drug-induced apoptosis [17–23]. Furthermore, the p53 inhibitor Pifithrin-α (PFT-α) protected otic cells from ototoxicity [24, 25]. However, among all these strategies to combat ototoxicity, only one drug, STS, has only very recently been approved by the Food and Drug Administration (FDA) as a treatment regimen to prevent cisplatin-induced ototoxicity. The efficacy of STS was analyzed in two clinical trials involving pediatric cancer patients who received cisplatin-based chemotherapy. In these trials, patients who received systemic STS administration had a 29% and 14% lower risk of hearing loss, respectively [17, 26]. However, it was also demonstrated that STS binds to cisplatin with high affinity, thereby inactivates it and thus can lower the chemotherapeutic effect of cisplatin at the tumor site after systemic administration [27]. Therefore, there is a strong need for additional novel inner ear-protective treatment options for patients undergoing ototoxic drug treatment.

Based on a previous study from our group that demonstrated anti-ototoxic effects by ectopic expression of the anti-apoptotic gene *BCL-XL*, we now tested anti-apoptotic small molecules other than the ones already tested in the context of ototoxicity, including Emricasan, and compared their effects to treatment with the published compounds Z-DEVD-FMK and PFT-α [28]. In a series of experiments conducted on various auditory cell types, it was demonstrated that the organic compound Emricasan is a highly effective small molecule for the prevention of apoptosis, through the inhibition of caspases. It significantly reduced cisplatin- and neomycin-induced cytotoxicity and demonstrated an even stronger effect than STS, particularly in neomycin-treated cells.

## Material and methods

The cell culture of HEI-OC1 and phoenix auditory cells as well as the preparation and cultivation of primary SGNs is described in greater detail in the Supplementary Material and Methods. To analyze cytotoxicity of the cochlear cells upon cisplatin or neomycin treatment, different parameters were determined, such as caspase-3/-7 activity, dead-cell protease activity, DNA fragmentation, ROS generation, mitochondrial membrane potential, Annexin V positivity, and cell morphology. The detailed protocol for each assay is described in the Supplementary Material and Methods.

### Statistical analyses

Data are displayed as mean ± standard deviation and were statistically analyzed using the GraphPad Prism 5 software (Dotmatics, Boston, US). One-way analysis of variance, followed by Dunnett’s post-hoc test, was used to compare multiple groups. A *p*-value ≤ 0.05 (*) was considered statistically significant, *p* ≤ 0.01 (**) was considered highly significant, and *p* ≤ 0.001 (***) was considered extremely significant. Differences that were not statistically significant were indicated by “ns.” The sample size (n) was stated in each figure legend. In the cell death assay, staining with Annexin V and DAPI was employed to statistically compare the Annexin V^+^/DAPI^+^ cells. Conversely, in the assay to determine the mitochondrial membrane potential, the percentage of JC-1 monomers was statistically compared.

## Results

### General caspase inhibitors are highly efficient anti-apoptotic small molecules in cisplatin-treated HEI-OC1 cells

**Fig. 1:**
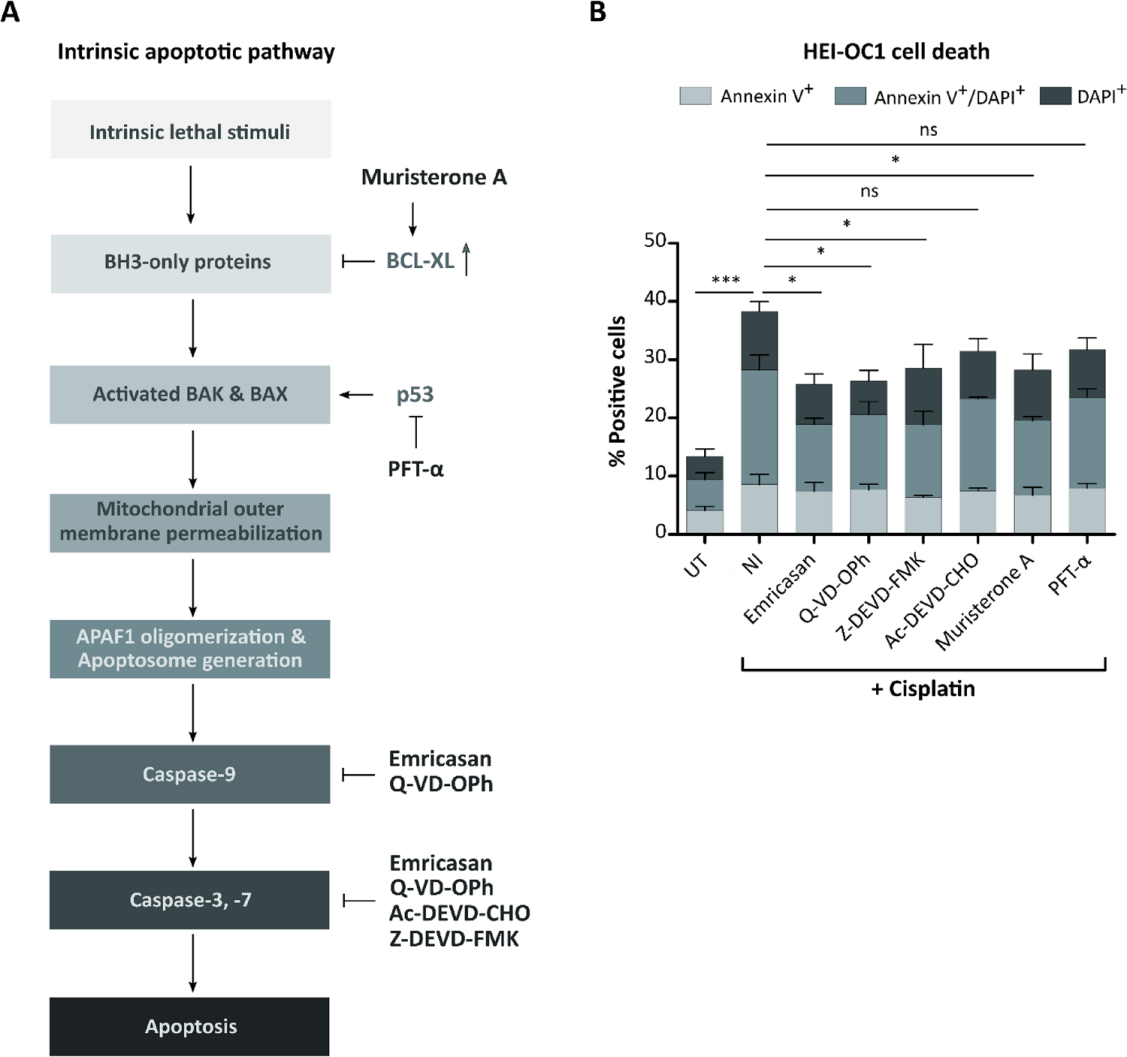
General caspase inhibitors Emricasan and Q-VD-OPh strongly alleviate cisplatin-mediated cytotoxicity in HEI-OC1 cells. **A** Schematic overview of the intrinsic apoptotic pathway, highlighting the targets of the anti-apoptotic small molecules employed in this study. The pathway is initiated by intrinsic lethal stimuli, which activate BH3-only proteins that activate the pro-apoptotic proteins BAK and BAX. BCL-XL, an anti-apoptotic protein, can prevent the continuation of the apoptotic pathway at this step. If p53 is involved in the apoptotic pathway, it activates pro-apoptotic proteins, leading to pore formation in the outer mitochondrial membrane, release of cytochrome c, and generation of the apoptosome with APAF1 (Apoptotic protease activating factor 1) and caspase-9. This initiates a cascade of caspase activation, followed by the final initiation of apoptosis. Muristerone A increases BCL-XL expression, while PFT-α inhibits p53. The general caspase inhibitors Emricasan and Q-VD-OPh can inhibit caspase-9 as well as the effector caspases caspase-3 and caspase-7, whereas Ac-DEVD-CHO and Z-DEVD-FMK specifically inhibit caspase-3 and caspase-7. **B** Annexin V and DAPI staining were used to analyze cell death at 72 h in cisplatin-treated (5 µM) and untreated (UT) HEI-OC1 cells. The performance of no inhibitor (NI) control cells was compared to cells to which the anti-apoptotic small molecules had been co-administered with the cisplatin. *N* = 3 independent experiments. The data in Fig. 1B are presented as mean ± standard deviation (SD) (*P* ≤ 0.05 (*), and *P* ≤ 0.001 (***), determined using one-way ANOVA together with Dunnett’s post-hoc test)

Since the intrinsic apoptotic pathway is activated in cisplatin-treated cochlear cells, we screened small molecules that specifically interfere with this pathway for their suitability to serve as drugs for preventing or ameliorating cisplatin-induced ototoxicity (Fig. 1A) [14, 15]. In particular, we tested the caspase inhibitors Emricasan, Q-VD-OPh, and Ac-DEVD-CHO, as well as the BCL-XL-activating compound Muristerone A, in addition to the reference small molecules Z-DEVD-FMK and PFT-α, which were previously demonstrated to prevent cisplatin-induced apoptosis. To determine if the selected anti-apoptotic small molecules can prevent otic cells from cisplatin-induced cytotoxicity, we evaluated their effectiveness in vitro using the established HEI-OC1 cell line. HEI-OC1 cells are derived from the organ of Corti of the transgenic Immortomouse™ and express markers of supporting and HCs, suggesting them as a potential progenitor of both cell types [29]. Moreover, HEI-OC1 cells are sensitive to ototoxic drugs, making them a suitable HC-like in vitro model for investigating strategies to prevent ototoxicity [30].

Application of the selected drugs to HEI-OC1 cells at pre-defined doses did not evoke any signs of cytotoxicity, as indicated by comparable cell death rates between untreated (UT) and small molecule-treated cells (Supplementary Fig. 1A). Therefore, these concentrations were used for all further experiments. To analyze the functionality of the caspase inhibitors, we induced apoptosis through cisplatin treatment and measured caspase-3/-7 activity to determine if the small molecules can reduce this activity in cisplatin-treated HEI-OC1 cells (Supplementary Fig. 1B). All tested caspase inhibitors could significantly reduce caspase-3/-7 activity and thus apoptosis, which was also reflected in reduced cell death compared to the cisplatin-treated no inhibitor (NI) control (Fig. 1B). For the cell death assay, cells were stained with Annexin V and DAPI to determine early apoptotic (Annexin V^+^), late apoptotic/dead (Annexin V^+^/DAPI^+^) and dead (DAPI^+^) HEI-OC1 cells. For Muristerone A, the reported feature of increased *BCL-XL* expression was confirmed by flow cytometry (Supplementary Fig. 1C), which contributed to the therapeutic effect of lower percentages of HEI-OC1 cells affected by cisplatin-induced cell death (Fig. 1B). PFT-α could reduce p53 phosphorylation seen in NI cells (Supplementary Fig. 1D and E) and could also lower cell death rates upon cisplatin treatment (Fig. 1B).

In summary, all tested anti-apoptotic small molecules showed molecular activity, and although they targeted different apoptosis mediators, they all achieved a therapeutic effect by reducing cisplatin-induced cytotoxicity in HEI-OC1 cells. However, since the general caspase inhibitors demonstrated the tendency of a slightly stronger reduction of cell death, we next focused on this group of inhibitors.

### Emricasan alone is sufficient to prevent cisplatin-induced cytotoxicity in HEI-OC1 cells

The previous experiments showed that general caspase inhibitors may be promising compounds for reducing cisplatin-induced toxicity in otic cells. Since Emricasan has already been tested in clinical trials to treat certain liver diseases and was considered safe and well-tolerated, we focused on this general caspase inhibitor in the following experiments [31–33].

**Fig. 2:**
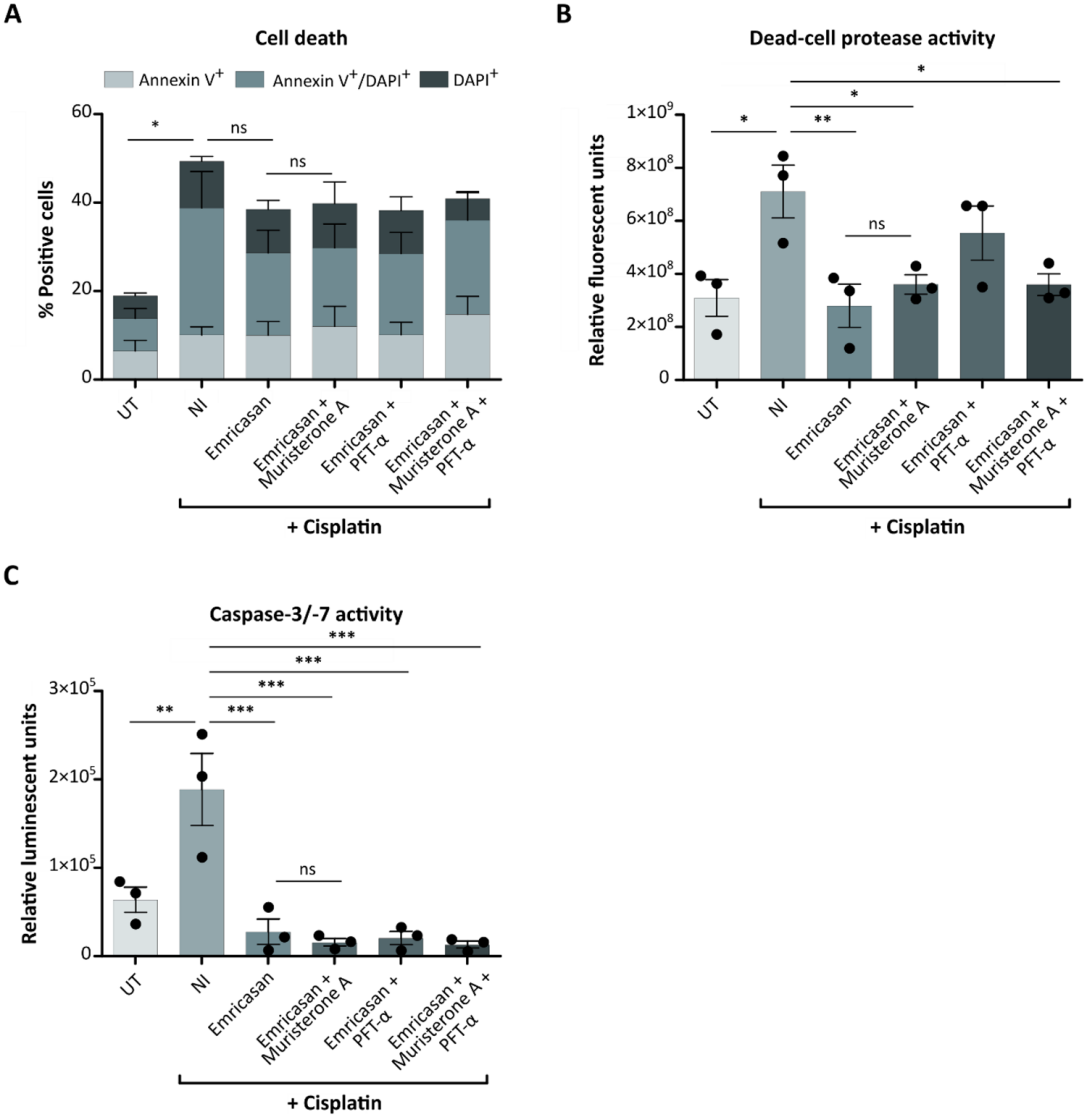
The combination of anti-apoptotic small molecules does not result in an additive effect to reduce cisplatin-induced cytotoxicity in HEI-OC1 cells. HEI-OC1 cells were left untreated (UT) or treated with cisplatin in the absence (No inhibitor, NI) or presence of the depicted small molecule combinations. Emricasan was combined with Muristerone A, PFT-α, or both small molecules and compared to the performance of NI control cells and cisplatin-treated Emricasan-only cells. **A** Percentage of early apoptotic (Annexin V^+^), late apoptotic/dead (Annexin V^+^/DAPI^+^), and dead (DAPI^+^) cells in the different HEI-OC1 cultures. **B** Analysis of the dead-cell protease activity using the ApoTox-Glo™ Triplex Assay. **C** Caspase-3/-7 activity determined using the ApoTox-Glo™ Triplex Assay. *N* = 3 independent experiments. The data is depicted as mean ± standard deviation (SD) (*P* ≤ 0.05 (*), *P* ≤ 0.01 (**), and *P* ≤ 0.001 (***), ns = non-significant, determined using one-way ANOVA together with Dunnett's post-hoc test). Cells were treated with 5 µM cisplatin for 72 h. The small molecules were added in the following concentrations: Emricasan = 10 µM, Muristerone A = 3 µM, PFT-α = 0.5 µM

First, we investigated whether combining Emricasan with other anti-apoptotic small molecules targeting different proteins in the intrinsic apoptotic pathway could lead to additive effects. Therefore, we evaluated the efficacy in reducing cytotoxicity in cisplatin-treated HEI-OC1 cell cultures of Emricasan in combination with Muristerone A or with PFT-α, as well as of all three anti-apoptotic small molecules co-applied (Fig. 2). Annexin V/DAPI co-staining revealed reduced cell death in cisplatin-treated cultures treated with different small molecule combinations compared to NI, even though this effect was not significant (Fig. 2A). Emricasan alone was as efficient at reducing apoptosis in cisplatin-treated cultures as were the different drug combinations, demonstrating no additive effect of the tested molecules. We additionally measured dead-cell protease (Fig. 2B) and caspase-3/-7 (Fig. 2C) activity in these cultures. These parameters were significantly elevated in cisplatin-treated NI cells compared to UT cells. All small molecule combinations achieved significantly reduced levels of dead-cell protease and caspase-3/-7 activity compared to NI cells. However, the combinations were not superior to the Emricasan treatment alone. Strikingly, Emricasan treatment not only achieved a highly significant effect, but resulted in similar (dead-cell protease activity) or even lower (caspase-3/-7 activity) levels of cytotoxicity markers than seen in UT cultures.

**Fig. 3:**
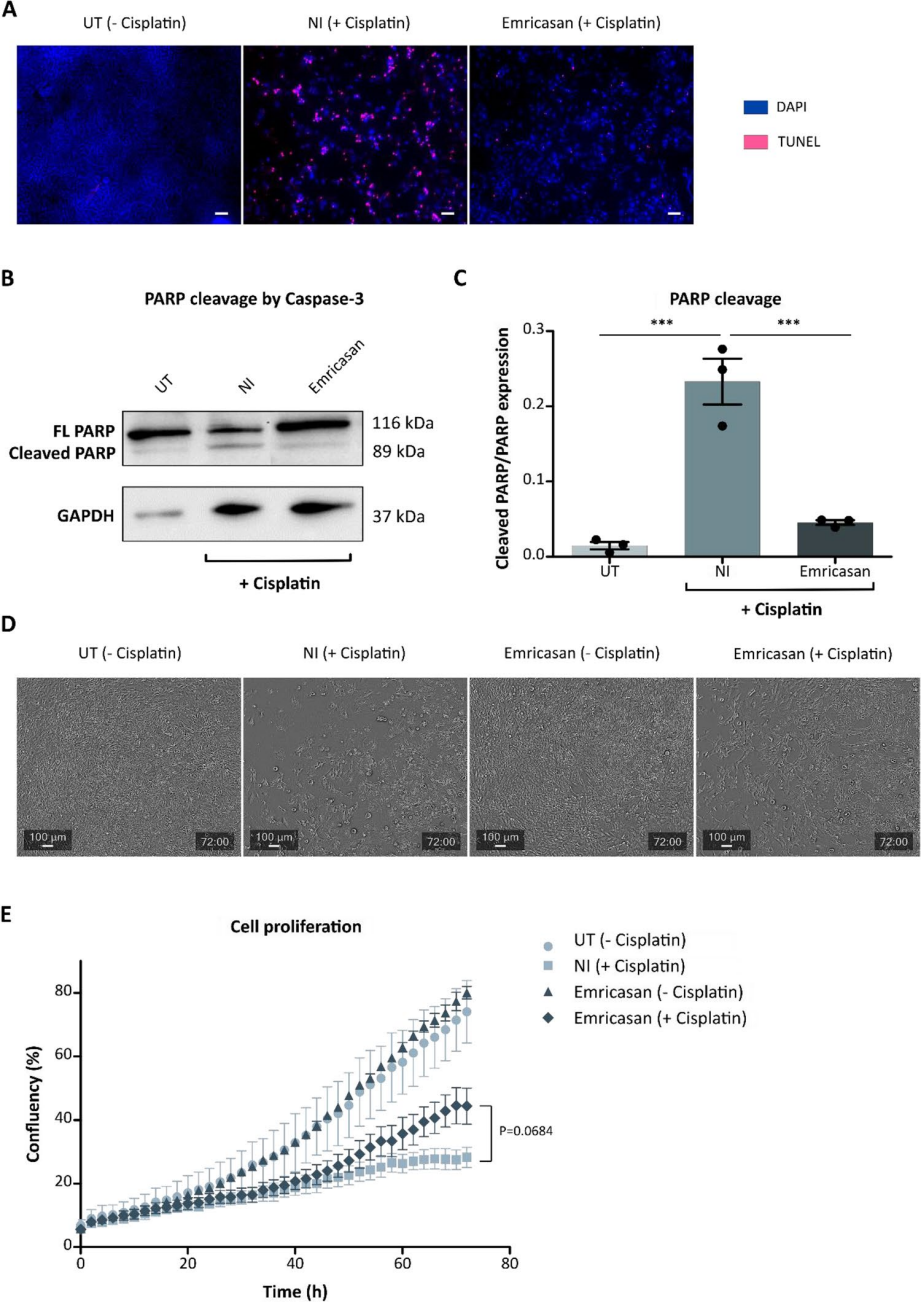
Emricasan increases the viability of cisplatin-treated HEI-OC1 cells as evidenced by reduced apoptosis and increased confluency. The effects of Emricasan treatment were characterized by comparing untreated (UT) control, cisplatin-treated without Emricasan (No inhibitor, NI), and Emricasan plus cisplatin co-treated HEI-OC1 cultures.** A** Representative pictures from TUNEL assay to investigate DNA fragmentation, indicated by TUNEL-positive (pink) cells. All samples were counterstained with DAPI (blue). Scale bar = 20 µm. **B** Western blot analysis to investigate the expression of full-length (FL) PARP (116 kDa) and cleaved PARP (89 kDa) induced by caspase-3. Endogenous GAPDH (37 kDa) levels served as loading control. **C** Quantification of western blot signals showing the ratio of cleaved PARP to full-length PARP. **D** Representative microscopy pictures from the different cultures. Scale bar = 100 µm. **E** Live-cell imaging results using the CellCyte X™ to determine cell confluency over 72 h upon the administration of cisplatin in the presence or absence of Emricasan compared to UT cells. *N* = 3 independent experiments. The data is depicted as mean ± standard deviation (SD) (*P* ≤ 0.001 (***), determined using one-way ANOVA together with Dunnett's post-hoc test). Cisplatin (5 µM) and Emricasan (10 µM) co-treatment was performed over 72 h

In conclusion, no additive effect was observed when combining Emricasan with different anti-apoptotic small molecules to prevent cisplatin-induced cytotoxicity. Therefore, we next characterized Emricasan's effects on apoptotic processes in greater detail by analyzing additional hallmarks of apoptosis beyond caspase-3/-7 activation and Annexin V positivity [34, 35]. As one stringent hallmark, fragmented DNA was detected in cisplatin-treated HEI-OC1 cells by TUNEL staining (Fig. 3A). Strikingly, the addition of Emricasan strongly reduced the extent of DNA fragmentation. As additional evidence that Emricasan effectively inhibits cisplatin-induced apoptosis in HEI-OC1 cells, its addition significantly reduced PARP cleavage by caspase-3 compared to NI cells (Fig. 3B and C). We also assessed the confluency and morphology of cisplatin-treated HEI-OC1 cultures in the presence and absence of Emricasan as an indicator of cell viability. For this, live-cell imaging was performed. After 72 h of incubation, a confluent cell layer was observed in the UT and Emricasan-only treated cells. Notably, cisplatin treatment resulted in more dead cells in the supernatant and less confluent cells in the NI cultures. However, adding Emricasan to cisplatin-treated HEI-OC1 cells resulted in a more confluent cell layer than in the NI control cells, indicating a partial prevention of the cisplatin-induced decrease in confluency (Fig. 3D). The quantification of live-cell imaging data revealed increased confluency over time, with approximately 80% confluency at 72 h in both UT and Emricasan-only cells (Fig. 3E). NI control cultures reached approximately 20% confluency after 72 h, while the corresponding Emricasan co-treated cells showed approximately 40% confluency.

In summary, Emricasan counteracted apoptotic hallmarks upon cisplatin administration in HEI-OC1 cells and increased cell confluency compared to NI cells.

### One-time administration of Emricasan achieves prolonged beneficial effects in cisplatin-treated HEI-OC1 cells

**Fig. 4:**
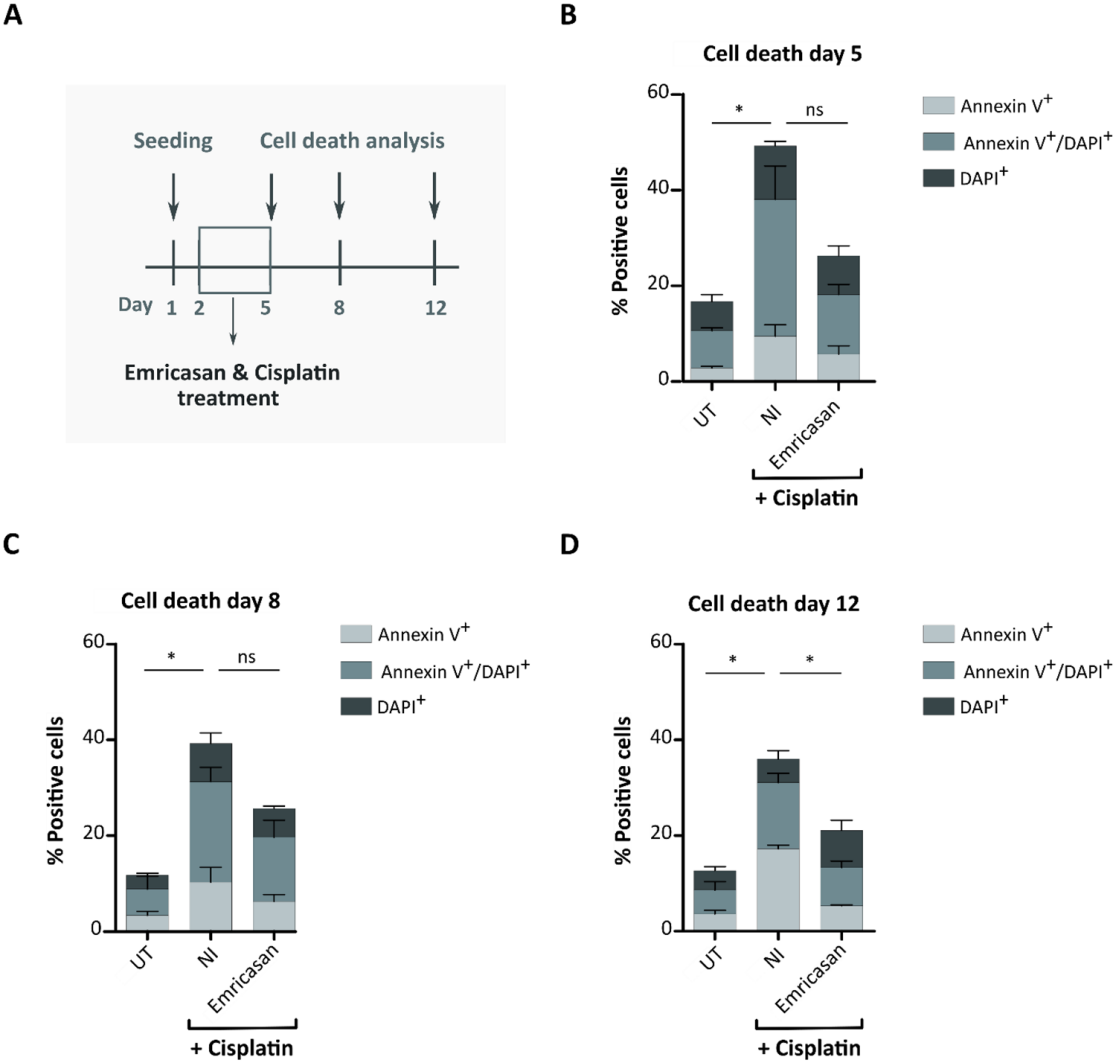
The anti-apoptotic effect of Emricasan in cisplatin-treated HEI-OC1 cells is prolonged. **A** Schematic overview of the experimental layout. The experiment started on day 1 with cell seeding, followed by Emricasan and cisplatin co-administration on day 2, with the drugs left on the cells until day 5. Cell death analyses were performed on days 5, 8, and 12. Untreated (UT) cells and cisplatin-only (No inhibitor, NI) treated cells served as controls. **B**–**D** Analysis of HEI-OC1 cell death by determination of the percentage of Annexin V^+^ (early apoptotic), Annexin V^+^/DAPI^+^ (late apoptotic/dead), and DAPI^+^ (dead) cells in untreated (UT), cisplatin only- (NI), and Emricasan co-treated cultures. *N* = 3 independent experiments. The data is depicted as mean ± standard deviation (SD) (P ≤ 0.05 (*), ns = non-significant, determined using one-way ANOVA together with Dunnett's post-hoc test). Cisplatin treatment: 5 µM for 72 h. Emricasan concentration: 10 µM

Small molecules, including Emricasan (50 min), often have a short half-life in blood plasma [36]. Thus, for potential future clinical application, it is crucial to determine if the effects of a single administration continue even after metabolization of the drug or if the drug needs to be reapplied. To address this question in vitro, we analyzed the effects of a single Emricasan administration to reduce cisplatin-induced cytotoxicity in HEI-OC1 cells at multiple time points (Fig. 4). For this, HEI-OC1 cells were seeded on day 1, and cisplatin and Emricasan were co-administered on day 2 for 72 h (Fig. 4A). Annexin V and DAPI staining was performed on days 5, 8, and 12 to determine apoptosis and cell death. The analysis on day 5 confirmed that Emricasan treatment reduced cell death related to cisplatin treatment in HEI-OC1 cells compared to NI cells (Fig. 4B). Importantly, the analyses on day 8 (Fig. 4C) and day 12 (Fig. 4D) likewise demonstrated lower percentages of Annexin V^+^/DAPI^+^ cells in Emricasan-treated as compared to NI cultures, indicating that, despite its short serum half-life, a single Emricasan application is sufficient to achieve a prolonged alleviation of cisplatin-induced toxicity in vitro.

### Emricasan significantly reduces cell death in cisplatin-treated phoenix auditory cells and primary SGN

**Fig. 5:**
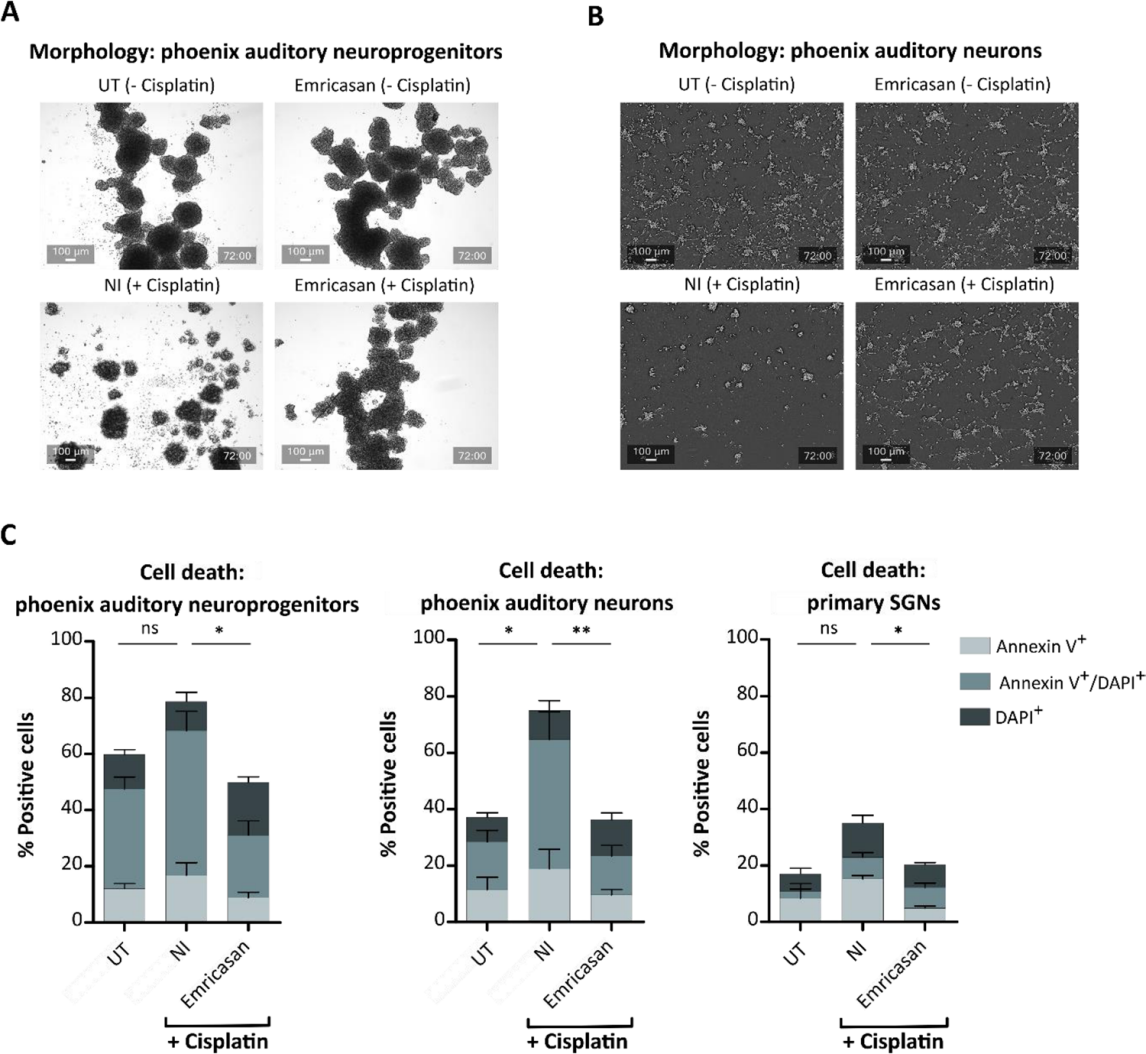
Emricasan significantly reduces cisplatin-induced toxicity in auditory neuronal cells. Representative photographs showing the morphology of phoenix auditory neuroprogenitors (**A**) and phoenix auditory neurons (**B**) in untreated (UT), cisplatin-treated (No inhibitor, NI), Emricasan-only, and cisplatin-treated Emricasan cells. Scale bar = 100 µm. **C** Analysis of the percentage of Annexin V^+^ (early apoptotic), Annexin V^+^/DAPI^+^ (late apoptotic/dead), and DAPI^+^ (dead) cells in phoenix auditory neuroprogenitor (left), phoenix auditory neuron (middle), and primary SGN (right) cultures that were untreated NI, Emricasan- and cisplatin co-treated. *N* = 3 independent experiments. The data is depicted as mean ± standard deviation (SD) (*P* ≤ 0.05 (*), *P* ≤ 0.01 (**), and ns = non-significant, determined using one-way ANOVA together with Dunnett's post-hoc test). Cisplatin treatment: 5 µM for phoenix auditory neuroprogenitors and 20 µM for phoenix auditory neurons or primary SGN for 72 h. Emricasan was applied at a dose of 10 µM

As cisplatin damages not only HCs but also SGNs, we next analyzed the effect of Emricasan in neuronal in vitro models, i.e., using phoenix auditory cells and primary rat SGN cultures. The phoenix auditory neuroprogenitors are derived from the A/J mouse cochlea and form spheres of different forms and sizes when cultured in a proliferation medium containing growth factors [37, 38]. In contrast to UT and Emricasan-only cells, which showed normal morphology, the NI culture showed less sphere formation, with more single or dead cells as well as more cell debris after 72 h of cisplatin treatment (Fig. 5A). Notably, cisplatin-treated cultures that received Emricasan appeared healthy and still formed spheres of varying sizes.

The phoenix auditory neuroprogenitors can differentiate into phoenix auditory neurons with a bipolar morphology (see UT and Emricasan-only cultures, Fig. 5B) upon replacement of the growth factors in the culture medium with neurotrophic factors. NI phoenix auditory neurons displayed fewer protrusions and more dead cells after cisplatin application. In contrast, cisplatin-treated phoenix auditory neurons co-treated with Emricasan exhibited a morphology similar to UT controls.

Emricasan’s beneficial effect was confirmed by cell death analyses on the phoenix auditory neuroprogenitor (Fig. 5C, left graph) and neuron cultures (Fig. 5C, middle graph). Strikingly, Emricasan significantly reduced the percentage of Annexin V^+^/DAPI^+^ cells in cisplatin-treated cultures to a level comparable to the UT control cells. Similar results were obtained in dissociated primary rat SGN cultures, in which Emricasan significantly reduced the percentage of Annexin V^+^ and/or DAPI^+^ cells compared to NI cultures (Fig. 5C, right graph).

These results suggest that Emricasan can protect neuronal cell types from cisplatin-induced cytotoxicity.

### Emricasan provides greater protection from cisplatin-induced toxicity than STS in neuronal cells

As the FDA approved the antioxidant STS as a preventative treatment regimen to protect pediatric cancer patients from cisplatin-induced ototoxic effects, we next aimed to compare the protective effects of Emricasan to STS. Since previous in vitro studies used a broad range of STS concentrations (15 µg/mL to 2 mg/mL), we included both a high (STS_high_; 2 mg/mL) and a low concentration of STS (STS_low_; 25 µg/mL). The high STS concentration reduced ROS generation in cisplatin-treated HEI-OC1 cells (Supplementary Fig. 2A). Moreover, STS_high_ achieved preservation of mitochondrial membrane potential in cisplatin-treated cultures as evidenced by the formation of JC-1 aggregates, which were reduced in NI counterparts, indicating healthy mitochondria and, thus, vital cells in the presence of STS_high_ (Supplementary Fig. 2B). Additionally, STS_high_ decreased cell death in HEI-OC1 cells following cisplatin treatment (Supplementary Fig. 2C). Emricasan also reduced ROS generation, increased the percentage of JC-1 aggregates, and enhanced the viability in cisplatin-treated HEI-OC1 cultures. In direct comparison, STS_high_ was significantly more effective, bringing almost all parameters tested to levels as observed in UT cells. Interestingly, the combination of Emricasan and STS_high_ was most effective in reducing the occurrence of Annexin V^+^ and/or DAPI^+^ cells.

**Fig. 6:**
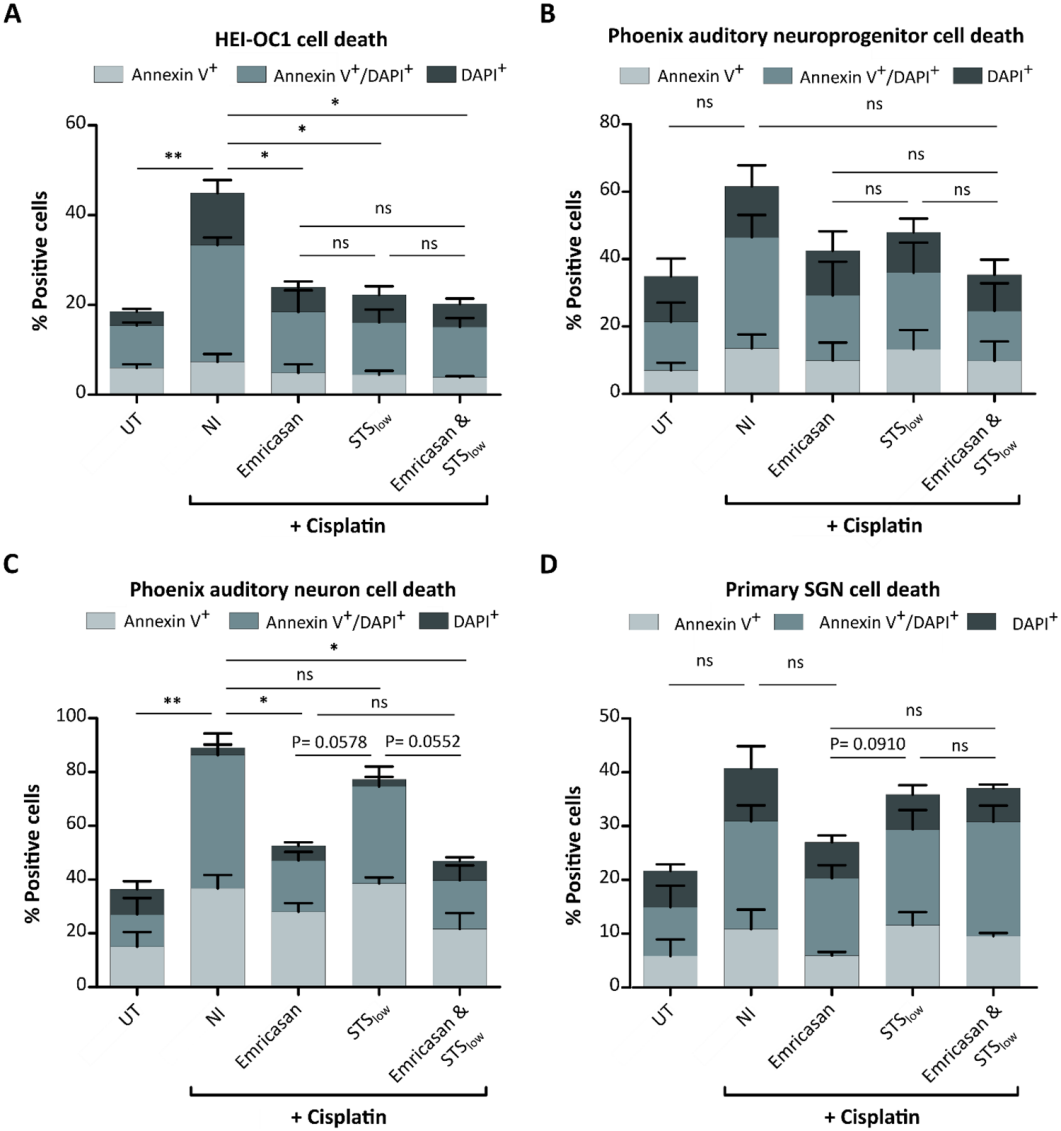
The reduction of cisplatin-induced cytotoxicity in neuronal cell types through Emricasan is superior to STS_low_. The indicated cell culture types were left untreated (UT), treated with cisplatin only (No inhibitor, NI) or co-treated with cisplatin and Emricasan (10 µM) and/or STS_low_ (25 µg/mL). **A**–**D** Cell death analysis based on determination of the percentage of early apoptotic (Annexin V^+^), late apoptotic/dead (Annexin V^+^/DAPI^+^), and dead (DAPI^+^) HEI-OC1 cells (**A**; 5 µM cisplatin), phoenix auditory neuroprogenitors (**B**; 5 µM cisplatin), phoenix auditory neurons (**C**; 20 µM cisplatin), and primary SGNs (**D**; 20 µM cisplatin). *N* = 3 independent experiments. The data is depicted as mean ± standard deviation (SD) (*P* ≤ 0.05 (*), *P* ≤ 0.01 (**), and ns = non-significant, determined using one-way ANOVA together with Dunnett’s post-hoc test)

Compared to STS_low_, Emricasan protected HEI-OC1 cells from cisplatin-induced death to a similar extent (Fig. 6A). However, in the phoenix auditory cells and primary SGN, Emricasan showed slightly stronger effects than STS_low_ (Fig. 6B–D). The combination of Emricasan and STS_low_ was slightly more effective at reducing cisplatin-induced toxicity in HEI-OC1 cells and phoenix auditory cells, suggesting a mild additive effect of the two small molecules.

In conclusion, the performance of STS is concentration-dependent, with STS_high_ slightly more effective at reducing cisplatin-induced toxicity in HEI-OC1 cells as compared to Emricasan, but with Emricasan being as effective as STS_low_ in HEI-OC1 cells and, importantly, being superior to STS_low_ in the neuronal cell cultures.

### The beneficial effect of Emricasan appears superior to that of STS in neomycin-treated cells

**Fig. 7:**
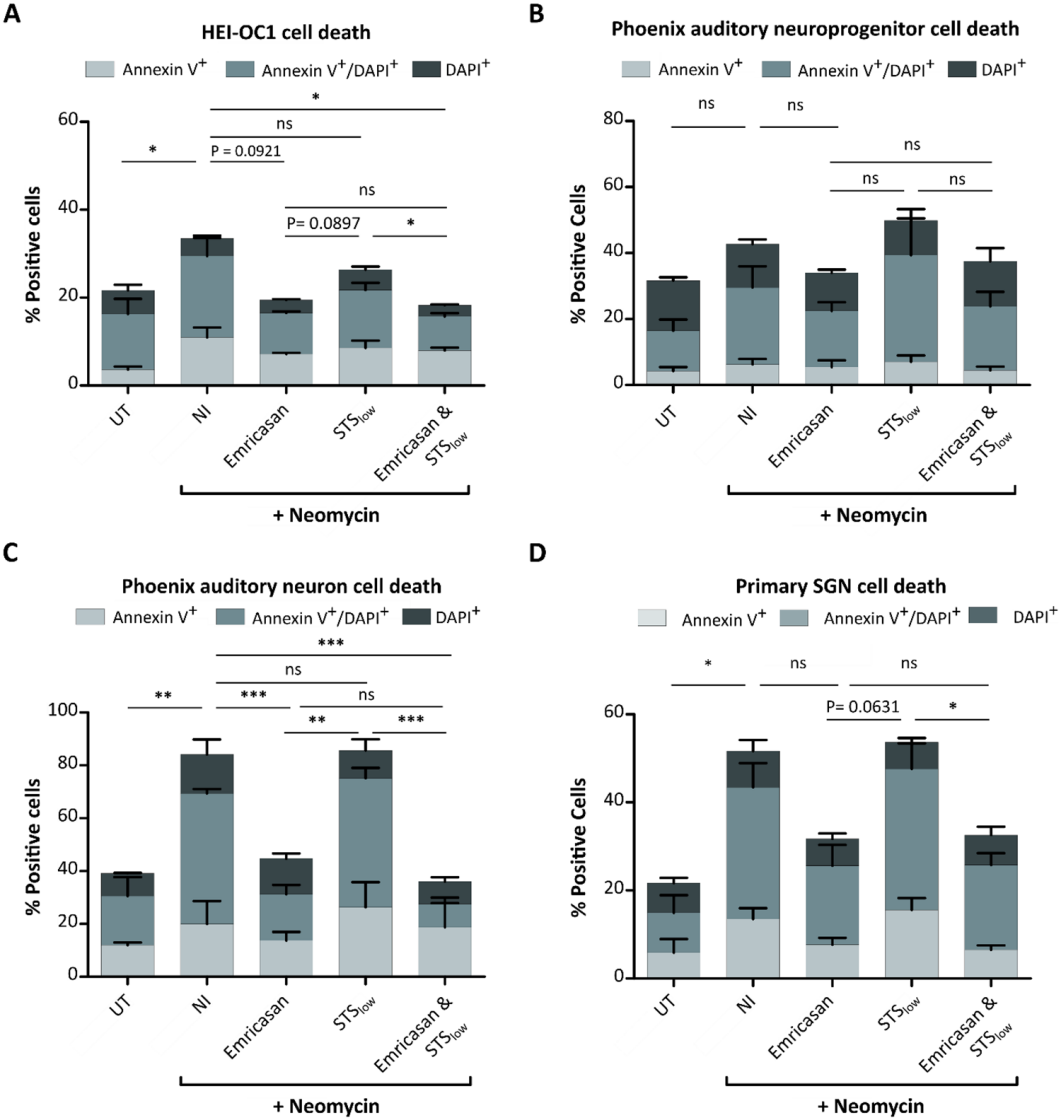
The reduction of neomycin-induced cytotoxicity in otic cell types through Emricasan is superior to STS_low_. The indicated cell culture types were left untreated (UT), treated with cisplatin only (No inhibitor, NI), or co-treated with cisplatin and Emricasan (10 µM) and/or STS_low_ (25 µg/mL). **A**–**D** Cell death analysis based on determination of the percentage of early apoptotic (Annexin V^+^), late apoptotic/dead (Annexin V^+^/DAPI^+^), and dead (DAPI^+^) HEI-OC1 cells (**A**), phoenix auditory neuroprogenitors (**B**), phoenix auditory neurons (**C**) and primary SGNs (**D**) upon treatment with 1 mM neomycin for 72 h (**A**–**C**) or 48 h (**D**). *N* = 3 independent experiments. The data is depicted as mean ± standard deviation (SD) (*P* ≤ 0.05 (*), *P* ≤ 0.01 (**), *P* ≤ 0.001 (***), and ns = non-significant, determined using one-way ANOVA together with Dunnett’s post-hoc test)

Because cisplatin and aminoglycosides, like neomycin, cause ototoxicity through similar mechanisms, such as increasing ROS generation and inducing apoptosis of cochlear cells, we next aimed to investigate whether Emricasan and/or STS could be beneficial for patients receiving aminoglycoside treatment. Hence, we tested Emricasan and both concentrations of STS either alone or in combination in different types of neomycin-treated cells (Fig. 7 and Supplementary Fig. 3). Strikingly, in HEI-OC1 cells (Fig. 7A), phoenix auditory neuroprogenitors (Fig. 7B), phoenix auditory neurons (Fig. 7C), and primary SGNs (Fig. 7D), Emricasan alone was found to be more effective at preventing cell death than STS_low_. Combining STS_low_ and Emricasan in HEI-OC1 cells and phoenix auditory neurons resulted in a slightly more efficient reduction of Annexin^+^/DAPI^+^ cells upon neomycin treatment than Emricasan alone.

Remarkably, the higher concentration of STS failed to reduce neomycin-induced toxicity in HEI-OC1 cells (Supplementary Fig. 3). Consequently, STS_high_ could not reduce ROS generation (Supplementary Fig. 3A) or maintain the mitochondrial membrane potential (Supplementary Fig. 3B). Moreover, the administration of STS_high_ did not reduce cell death rates in neomycin-treated HEI-OC1 cells (Supplementary Fig. 3C). In contrast, Emricasan significantly decreased the percentage of Annexin V^+^/DAPI^+^ cells compared to STS cultures, suggesting that Emricasan holds promise as a potential therapeutic drug candidate to prevent aminoglycoside-induced ototoxicity.

## Discussion

To our knowledge, we present the first study showing that Emricasan was effective as an anti-cytotoxic treatment under cisplatin and neomycin application in cochlear-derived cells. Beyond Emricasan, several other anti-apoptotic small molecules were evaluated here for their efficacy in preventing apoptosis in cisplatin-treated inner ear cells. The rationale for this study was based on the findings from a previous study, which suggested inhibition of apoptosis as an effective strategy for treating cisplatin-mediated hearing loss [28]. As suitable in vitro test systems, the murine cell lines HEI-OC1 and phoenix auditory cells, which represent HC- and SGN-like cells, and primary SGN cultures were used since they reflect the cell types that are most affected by ototoxic drug treatment within the cochlea.

The performance of Emricasan was compared to the recently approved anti-ototoxic drug STS. In neomycin-treated cells, Emricasan was more effective in preventing cytotoxicity than STS, regardless of the STS concentration. In cisplatin-treated cells, the effectiveness of Emricasan compared to STS depended on the STS concentration applied. However, compared to a lower STS concentration, Emricasan showed improved protection of neuronal cells from the toxic effects of cisplatin treatment.

STS acts as a ROS scavenger and has antioxidant properties. However, previous studies have also shown that STS binds to platinum with high affinity, leading to the inactivation of platinum-based chemotherapeutic agents, such as cisplatin [27]. This property is desired at off-target sites, such as the cochlea, but is not beneficial at the tumor site. As STS is administered intravenously to patients, it can potentially lower the anti-tumor effect of cisplatin. Our study further emphasized that the anti-cytotoxic effect of STS is not solely due to ROS scavenging, as STS was ineffective in preventing neomycin-induced cytotoxicity. Even at high concentrations, STS application failed to reduce the death of HEI-OC1 cells treated with neomycin. As neomycin does not contain platinum, STS likely does not bind and inactivate the antibiotic. Thus, the antioxidant property of STS may not be sufficient to protect otic cells from neomycin-induced cell death. In contrast, the anti-apoptotic properties of Emricasan resulted in efficient prevention of the toxic effects of cisplatin and neomycin in the employed auditory cells.

Previously, other small molecules with anti-apoptotic properties were tested to protect otic cells from cisplatin or aminoglycoside-induced toxicity. The tetracycline antibiotic minocycline can inhibit caspases and the release of cytochrome c following cisplatin or gentamicin treatment of HEI-OC1 cells and rat cochlea explant cultures or even after in vivo application to guinea pigs [39, 40]. The caspase-9 inhibitor Z-LEHD-FMK demonstrated anti-ototoxic effects and increased hair cell survival in guinea pigs treated with either cisplatin or gentamicin [22, 23]. Similarly, the caspase-3 inhibitor Z-DEVD-FMK protected inner ear cells from cisplatin-induced ototoxicity in guinea pigs [22]. The general caspase inhibitor Z-VAD-FMK also efficiently decreased the ototoxic effects in guinea pigs treated with gentamicin [22, 23]. Additionally, the impact of p53-induced apoptosis following cisplatin treatment was verified in rat cochlea explants, and the survival of HCs increased with the use of the p53 inhibitor PFT-α [24, 25]. Similar to these findings, the caspase-3 and p53 inhibitors also demonstrated some beneficial effects in reducing cisplatin-induced toxicity in HEI-OC1 cells in our hands. However, general caspase inhibitors showed slightly greater anti-cytotoxic effects, suggesting inhibition of multiple caspases is important to elicit a more complete inhibition of drug-induced cell death.

As the next step, in vivo experiments are required to investigate the potential benefits of Emricasan further. It is recommended that Emricasan is administered through local injection into the inner ear to minimize its possible interference with the anti-tumoral effect of cisplatin at the tumor site or the bactericidal effect of aminoglycosides when given systemically [41, 42]. Given that small molecules typically have a short half-life and that chemotherapy involves multiple rounds and antibiotic use covers several days, multiple injections may be required to achieve the desired therapeutic effect of Emricasan. However, multiple local injections of the small molecule may not be feasible, could be costly, and could lead to inflammation [43]. Therefore, an optimal approach would allow the small molecule to gradually reach the cochlea, specifically the HCs and SGNs. Such a temporally controlled distribution approach could be tested with hydrogels or a miniature osmotic pump for drug delivery [44–47]. Alternatively, it could be examined whether a systemic administration of Emricasan results in a sufficient concentration of the otoprotective agent within the inner ear. If this is the case, different administration time points during or after the cisplatin infusion/aminoglycoside intake could be analyzed in order to achieve the maximum otoprotective effect of Emricasan, while minimizing the interference with the ototoxic drug at the on-target site. When given systemically, it may be possible that rare symptoms such as headache, nausea, or dehydration, as observed in the clinical trials with Emricasan to fight certain liver diseases, might be observed in patients [32].

In conclusion, we here demonstrate that Emricasan exhibits a strong anti-cytotoxic effect in vitro in HEI-OC1 cells, phoenix auditory neurons, and primary SGN treated with cisplatin or neomycin. The effect was even superior to STS, the only anti-ototoxic drug approved for clinical use. Our next step is to test Emricasan in vivo to advance this approach toward a potential clinical application to protect patients from drug-induced ototoxicity.

### Supplementary information

Below is the link to the electronic supplementary material.

Supplementary file1 (PDF 663 KB)

## Data Availability

All data are available in the main text or the supplementary materials.

## References

[1] Arslan E, Orzan E, Santarelli R (1999) Global problem of drug-induced hearing loss. Ann NY Acad Sci 884:1–14. 10.1111/j.1749-6632.1999.tb00277.x 10842579 10.1111/j.1749-6632.1999.tb00277.x

[2] Lanvers-Kaminsky C, Am Z-D, Parfitt R, Ciarimboli G (2017) Drug-induced ototoxicity: mechanisms, pharmacogenetics, and protective strategies. Clin Pharmacol Ther 101:491–500. 10.1002/cpt.603 28002638 10.1002/cpt.603

[3] Kopke RD, Liu W, Gabaizadeh R et al (1997) Use of organotypic cultures of Corti’s organ to study the protective effects of antioxidant molecules on cisplatin-induced damage of auditory hair cells. Am J Otol 18:559–571 9303151

[4] Schacht J, Talaska AE, Rybak LP (2012) Cisplatin and aminoglycoside antibiotics: hearing loss and its prevention. Anat Rec (Hoboken) 295:1837–1850. 10.1002/ar.22578 23045231 10.1002/ar.22578 PMC3596108

[5] Kulik M, Mori T, Sugita Y, Trylska J (2018) Molecular mechanisms for dynamic regulation of N1 riboswitch by aminoglycosides. Nucleic Acids Res 46:9960–9970. 10.1093/nar/gky833 30239867 10.1093/nar/gky833 PMC6212780

[6] Brown A, Kumar S, Tchounwou PB (2019) Cisplatin-based chemotherapy of human cancers. J Cancer Sci Ther 11:97 32148661 PMC7059781

[7] Ganesan P, Schmiedge J, Manchaiah V et al (2018) Ototoxicity: a challenge in diagnosis and treatment. J Audiol Otol 22:59–68. 10.7874/jao.2017.00360 29471610 10.7874/jao.2017.00360 PMC5894487

[8] Cianfrone G, Pentangelo D, Cianfrone F et al (2011) Pharmacological drugs inducing ototoxicity, vestibular symptoms and tinnitus: a reasoned and updated guide. Eur Rev Med Pharmacol Sci 15:601–636 21796866

[9] Dammeyer P, Hellberg V, Wallin I et al (2014) Cisplatin and oxaliplatin are toxic to cochlear outer hair cells and both target thioredoxin reductase in organ of Corti cultures. Acta Otolaryngol 134:448–454. 10.3109/00016489.2013.879740 24702224 10.3109/00016489.2013.879740 PMC4025594

[10] Guthrie OW, Li-Korotky HS, Durrant JD, Balaban C (2008) Cisplatin induces cytoplasmic to nuclear translocation of nucleotide excision repair factors among spiral ganglion neurons. Hear Res 239:79–91. 10.1016/j.heares.2008.01.013 18329831 10.1016/j.heares.2008.01.013

[11] Dodson HC (1997) Loss and survival of spiral ganglion neurons in the guinea pig after intracochlear perfusion with aminoglycosides. J Neurocytol 26:541–556. 10.1023/a:1015434524040 9350806 10.1023/a:1015434524040

[12] Schuknecht HF, Montandon P (1970) Pathology of the ear in pneumococcal meningitis. Arch Klin Exp Ohren Nasen Kehlkopfheilkd 195:207–225. 10.1007/BF00302950 5435957 10.1007/BF00302950

[13] Itoh T, Terazawa R, Kojima K et al (2011) Cisplatin induces production of reactive oxygen species via NADPH oxidase activation in human prostate cancer cells. Free Radic Res 45:1033–1039. 10.3109/10715762.2011.591391 21682664 10.3109/10715762.2011.591391

[14] Bragado P, Armesilla A, Silva A, Porras A (2007) Apoptosis by cisplatin requires p53 mediated p38α MAPK activation through ROS generation. Apoptosis 12:1733–1742. 10.1007/s10495-007-0082-8 17505786 10.1007/s10495-007-0082-8

[15] Denamur S, Tyteca D, Marchand-Brynaert J et al (2011) Role of oxidative stress in lysosomal membrane permeabilization and apoptosis induced by gentamicin, an aminoglycoside antibiotic. Free Radic Biol Med 51:1656–1665. 10.1016/j.freeradbiomed.2011.07.015 21835240 10.1016/j.freeradbiomed.2011.07.015

[16] Nwibo DD, Levi CA, Nwibo MI (2015) Small molecule drugs; down but not out: a future for medical research and therapeutics. IOSR Dent Med Sci 14:70–77. 10.9790/0853-14117077 10.9790/0853-14117077

[17] Brock PR, Maibach R, Childs M et al (2018) Sodium thiosulfate for protection from cisplatin-induced hearing loss. N Engl J Med 378:2376–2385. 10.1056/nejmoa1801109 29924955 10.1056/nejmoa1801109 PMC6117111

[18] Choe W-T, Chinosornvatana N, Chang KW (2004) Prevention of cisplatin ototoxicity using transtympanic N-acetylcysteine and lactate. Otol Neurotol 25:910–915. 10.1097/00129492-200411000-00009 15547419 10.1097/00129492-200411000-00009

[19] Aladag I, Guven M, Songu M (2016) Prevention of gentamicin ototoxicity with N-acetylcysteine and vitamin A. J Laryngol Otol 130:440–446. 10.1017/S0022215116000992 27095551 10.1017/S0022215116000992

[20] Hill GW, Morest DK, Parham K (2008) Cisplatin-induced ototoxicity: effect of intratympanic dexamethasone injections. Otol Neurotol 29:1005–1011. 10.1097/MAO.0b013e31818599d5 18716567 10.1097/MAO.0b013e31818599d5 PMC2720789

[21] Murphy D, Daniel SJ (2011) Intratympanic dexamethasone to prevent cisplatin ototoxicity: a guinea pig model. Otolaryngol Head Neck Surg 145:452–457. 10.1177/0194599811406673 21521888 10.1177/0194599811406673

[22] Wang J, Ladrech S, Pujol R et al (2004) Caspase inhibitors, but not c-Jun NH 2-terminal kinase inhibitor treatment, prevent cisplatin-induced hearing loss. Cancer Res 64:9217–9224. 10.1158/0008-5472.CAN-04-1581 15604295 10.1158/0008-5472.CAN-04-1581

[23] Okuda T, Sugahara K, Takemoto T et al (2005) Inhibition of caspases alleviates gentamicin-induced cochlear damage in guinea pigs. Auris Nasus Larynx 32:33–37. 10.1016/j.anl.2004.11.006 15882823 10.1016/j.anl.2004.11.006

[24] Zhang M, Liu W, Ding D, Salvi R (2003) Pifithrin-α supresses p53 and protects cochlear and vestibular hair cells from cisplatin-induced apoptosis. Neuroscience 120:191–205. 10.1016/S0306-4522(03)00286-0 12849752 10.1016/S0306-4522(03)00286-0

[25] Denamur S, Boland L, Beyaert M et al (2016) Subcellular mechanisms involved in apoptosis induced by aminoglycoside antibiotics: Insights on p53, proteasome and endoplasmic reticulum. Toxicol Appl Pharmacol 309:24–36. 10.1016/j.taap.2016.08.020 27568863 10.1016/j.taap.2016.08.020

[26] Freyer DR, Chen L, Krailo MD et al (2017) Effects of sodium thiosulfate versus observation on development of cisplatin-induced hearing loss in children with cancer (ACCL0431): a multicentre, randomised, controlled, open-label, phase 3 trial. Lancet Oncol 18:63–74. 10.1016/S1470-2045(16)30625-8 27914822 10.1016/S1470-2045(16)30625-8 PMC5520988

[27] Uozumi J, Ishizawa M, Lwamoto Y, Baba T (1984) Sodium thiosulfate inhibits cis-diamminedichloroplatinum (II) activity. Cancer Chemother Pharmacol 13:82–85. 10.1007/BF00257119 6540631 10.1007/BF00257119

[28] Nassauer L, Staecker H, Huang P et al (2024) Protection from cisplatin-induced hearing loss with lentiviral vector-mediated ectopic expression of the anti-apoptotic protein BCL-XL. Mol Ther Nucleic Acids 35:102157. 10.1016/j.omtn.2024.102157 38450280 10.1016/j.omtn.2024.102157 PMC10915631

[29] Kalinec GM, Webster P, Lim DJ, Kalinec F (2003) A cochlear cell line as an in vitro system for drug ototoxicity screening. Audiol Neurootol 8:177–189. 10.1159/000071059 12811000 10.1159/000071059

[30] Kalinec GM, Thein P, Park C, Kalinec F (2016) HEI-OC1 cells as a model for investigating drug cytotoxicity. Hear Res 335:105–117. 10.1016/j.heares.2016.02.019 26930622 10.1016/j.heares.2016.02.019

[31] Lekakis V, Cholongitas E (2022) The impact of emricasan on chronic liver diseases: current data. Clin J Gastroenterol 15:271–285. 10.1007/s12328-021-01585-2 35000120 10.1007/s12328-021-01585-2

[32] Shiffman M, Freilich B, Vuppalanchi R et al (2019) Randomised clinical trial: emricasan versus placebo significantly decreases ALT and caspase 3/7 activation in subjects with non-alcoholic fatty liver disease. Aliment Pharmacol Ther 49:64–73. 10.1111/apt.15030 30430605 10.1111/apt.15030 PMC6587784

[33] Harrison SA, Goodman Z, Jabbar A et al (2020) A randomized, placebo-controlled trial of emricasan in patients with NASH and F1–F3 fibrosis. J Hepatol 72:816–827. 10.1016/j.jhep.2019.11.024 31887369 10.1016/j.jhep.2019.11.024

[34] Jiang M, Qi L, Li L, Li Y (2020) The caspase-3/GSDME signal pathway as a switch between apoptosis and pyroptosis in cancer. Cell Death Discov 6:112. 10.1038/s41420-020-00349-0 33133646 10.1038/s41420-020-00349-0 PMC7595122

[35] Vermes I, Haanen C, Steffens-Nakken H, Reutelingsperger C (1995) A novel assay for apoptosis Flow cytometric detection of phosphatidylserine early apoptotic cells using fluorescein labelled expression on Annexin V. J Immunol Methods 184:39–51. 10.1016/0022-1759(95)00072-i 7622868 10.1016/0022-1759(95)00072-i

[36] Hoglen NC, Chen LS, Fisher CD et al (2004) Characterization of IDN-6556 (3-{2-(2-tert-Butyl-phenylaminooxalyl)-amino]-propionylamino}-4-oxo-5-(2,3,5, 6-tetrafluoro-phenoxy)-pentanoic acid): a liver-targeted caspase inhibitor. J Pharmacol Exp Ther 309:634–640. 10.1124/jpet.103.062034 14742742 10.1124/jpet.103.062034

[37] Rousset F, Schmidbauer D, Fink S et al (2022) Phoenix auditory neurons as 3R cell model for high throughput screening of neurogenic compounds. Hear Res 414:108391. 10.1016/j.heares.2021.108391 34844170 10.1016/j.heares.2021.108391

[38] Rousset F, Kokje VBC, Sipione R et al (2020) Intrinsically self-renewing neuroprogenitors from the A/J mouse spiral ganglion as virtually unlimited source of mature auditory neurons. Front Cell Neurosci 14:395. 10.3389/fncel.2020.599152 33362466 10.3389/fncel.2020.599152 PMC7761749

[39] Lee CK, Shin JI, Cho YS (2011) Protective effect of minocycline against cisplatin-induced ototoxicity. Clin Exp Otorhinolaryngol 4:77–82. 10.3342/ceo.2011.4.2.77 21716954 10.3342/ceo.2011.4.2.77 PMC3109331

[40] Corbacella E, Lanzoni I, Ding D et al (2004) Minocycline attenuates gentamicin induced hair cell loss in neonatal cochlear cultures. Hear Res 197:11–18. 10.1016/j.heares.2004.03.012 15504599 10.1016/j.heares.2004.03.012

[41] Nguyen TN, Park JS (2023) Intratympanic drug delivery systems to treat inner ear impairments. J Pharm Investig 53:93–118 10.1007/s40005-022-00586-8

[42] Yildiz E, Gadenstaetter AJ, Gerlitz M et al (2023) Investigation of inner ear drug delivery with a cochlear catheter in piglets as a representative model for human cochlear pharmacokinetics. Front Pharmacol 14:1062379. 10.3389/fphar.2023.1062379 36969846 10.3389/fphar.2023.1062379 PMC10034346

[43] Gehrke M, Sircoglou J, Gnansia D et al (2016) Ear cubes for local controlled drug delivery to the inner ear. Int J Pharm 509:85–94. 10.1016/j.ijpharm.2016.04.003 27050866 10.1016/j.ijpharm.2016.04.003

[44] El Kechai N, Bochot A, Huang N et al (2015) Effect of liposomes on rheological and syringeability properties of hyaluronic acid hydrogels intended for local injection of drugs. Int J Pharm 487:187–196. 10.1016/j.ijpharm.2015.04.019 25882015 10.1016/j.ijpharm.2015.04.019

[45] Borkholder DA, Zhu X, Frisina RD (2014) Round window membrane intracochlear drug delivery enhanced by induced advection. J Control Release 174:171–176. 10.1016/j.jconrel.2013.11.021 24291333 10.1016/j.jconrel.2013.11.021 PMC3925065

[46] Engleder E, Honeder C, Klobasa J et al (2014) Preclinical evaluation of thermoreversible triamcinolone acetonide hydrogels for drug delivery to the inner ear. Int J Pharm 471:297–302. 10.1016/j.ijpharm.2014.05.057 24907595 10.1016/j.ijpharm.2014.05.057 PMC4088987

[47] Al-Mahallawi AM, Khowessah OM, Shoukri RA (2014) Nano-transfersomal ciprofloxacin loaded vesicles for non-invasive trans-tympanic ototopical delivery: In-vitro optimization, ex-vivo permeation studies, and in-vivo assessment. Int J Pharm 472:304–314. 10.1016/j.ijpharm.2014.06.041 24971692 10.1016/j.ijpharm.2014.06.041

