# Supplementary material for: The caspase-inhibitor Emricasan efficiently counteracts cisplatin- and neomycin-induced cytotoxicity in cochlear cells
Source: J Mol Med (Berl). 2024 Aug 7;102(9):1163–74. doi: 10.1007/s00109-024-02472-2 (PMC11358181; doi:10.1007/s00109-024-02472-2)
Supplement: Supplementary file 1 — Supplementary file1 (PDF 663 KB) [file 109_2024_2472_MOESM1_ESM.pdf]

Journal of Molecular Medicine

The caspase-inhibitor Emricasan efficiently counteracts cisplatin- and neomycin-induced cytotoxicity in cochlear cells

Larissa Nassauer[1], Juliane W. Schott[1], Jennifer Harre[2,3], Athanasia Warnecke[2,3], Michael Morgan[1], Melanie Galla[1*], and Axel Schambach[1,4*]

[1] Institute of Experimental Hematology, Hannover Medical School, Hannover, 30625, Germany.

[2] Department of Otorhinolaryngology, Head and Neck Surgery, Hannover Medical School, 30625 Hannover, Germany.

[3] Cluster of Excellence "Hearing4all", Hannover Medical School, 30625 Hannover, Germany.

[4] Division of Hematology/Oncology, Boston Children's Hospital, Harvard Medical School, Boston, MA, 02115, USA.

* To whom correspondence should be addressed:

Axel Schambach, MD, PhD
Institute of Experimental Hematology
Hannover Medical School
Carl-Neuberg-Strasse 1
D-30625 Hannover, Germany
Tel: +49 511 532 6067
Fax: +49 511 532 6068
Email: Schambach.Axel@mh-hannover.de

Melanie Galla, PhD
Institute of Experimental Hematology
Hannover Medical School
Carl-Neuberg-Strasse 1
D-30625 Hannover, Germany
Tel: +49 511 532 5102
Email: Galla.Melanie@mh-hannover.de

Supplementary Figures

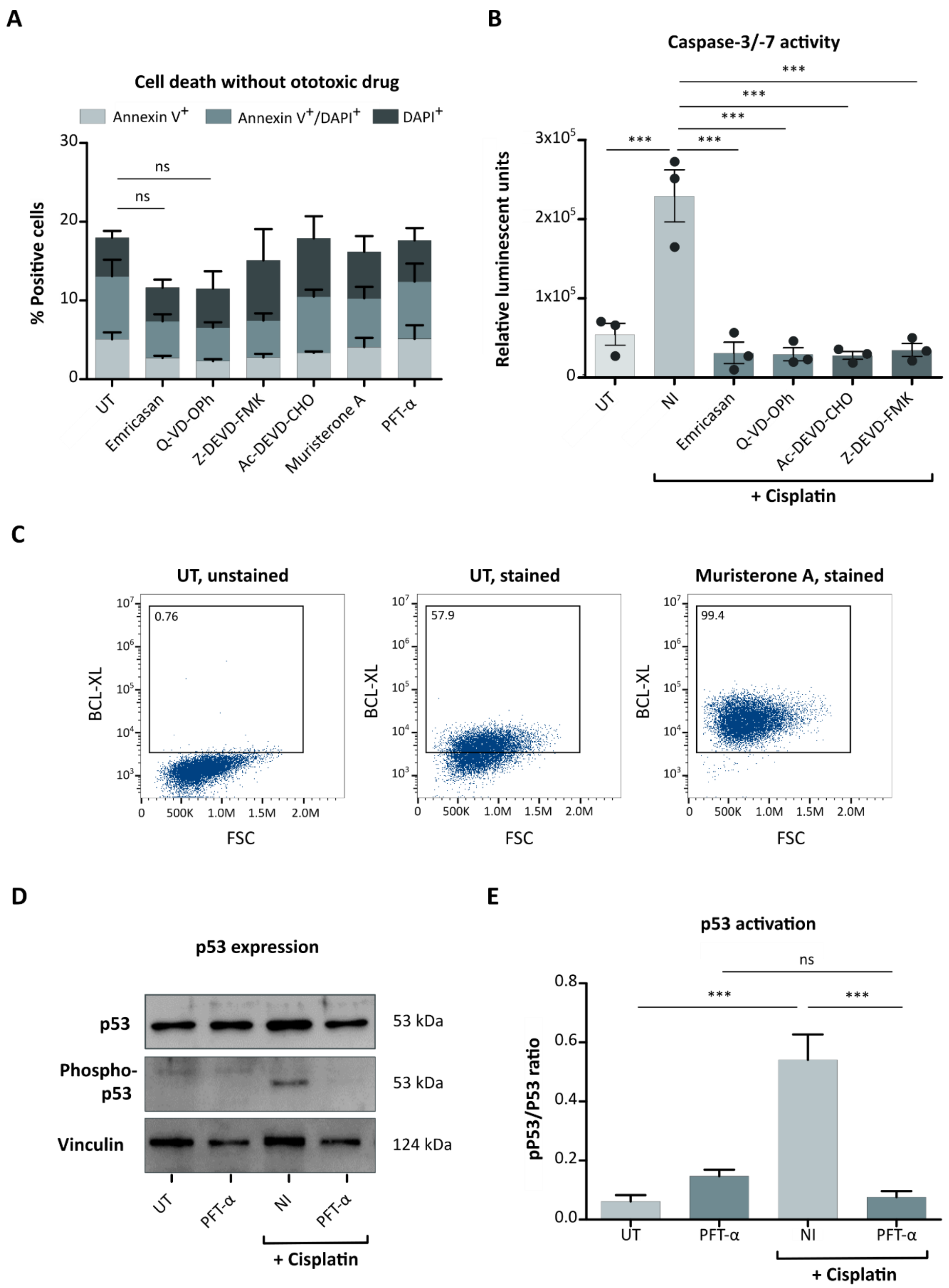

Supplementary Fig. 1 The selected anti-apoptotic small molecules are functional and well-tolerated in HEI-OC1 cells

A Viability analysis of untreated (UT) and small molecule-treated HEI-OC1 cultures by Annexin V and DAPI staining. **B** Investigation of caspase-3/-7 activity using the ApoTox-Glo™ Triplex Assay in UT and cisplatin-treated (No inhibitor, NI) HEI-OC1 cells, as well as in HEI-OC1 cells co-treated with cisplatin and a caspase inhibitor. **C** Representative flow cytometry plots showing BCL-XL expression in untreated and Muristerone A-treated HEI-OC1 cells detected by intracellular staining using an BCL-XL antibody, in comparison to unstained cells. **D** Western blot analysis to determine total p53 (53 kDa) and phosphorylated p53 protein (53 kDa) in UT, NI, and PFT-α- and cisplatin co-treated HEI-OC1 cells. Endogenous vinculin (124 kDa) protein served as loading control. **E** Quantification of P53 and phosphorylated P53 (pP53) protein upon western blot analysis showing the ratio of pP53 to p53 in untreated, PFT-α, and cisplatin-treated HEI-OC1 cells. $N = 3$ biological replicates. The data in (A), (B) and (E) is depicted as mean ± standard deviation (SD) ($P \leq 0.001$ (***) and ns = non-significant, determined using one-way ANOVA together with Dunnett's post-hoc test). Cisplatin treatment: 5 µM for 72 h. Small molecule concentrations: Emricasan = 10 µM, Q-VD-OPh =20 µM, Z-DEVD-FMK = 20 µM, Ac-DEVD-CHO = 100 nM, Muristerone A = 3 µM, PFT-α = 0.5 µM.

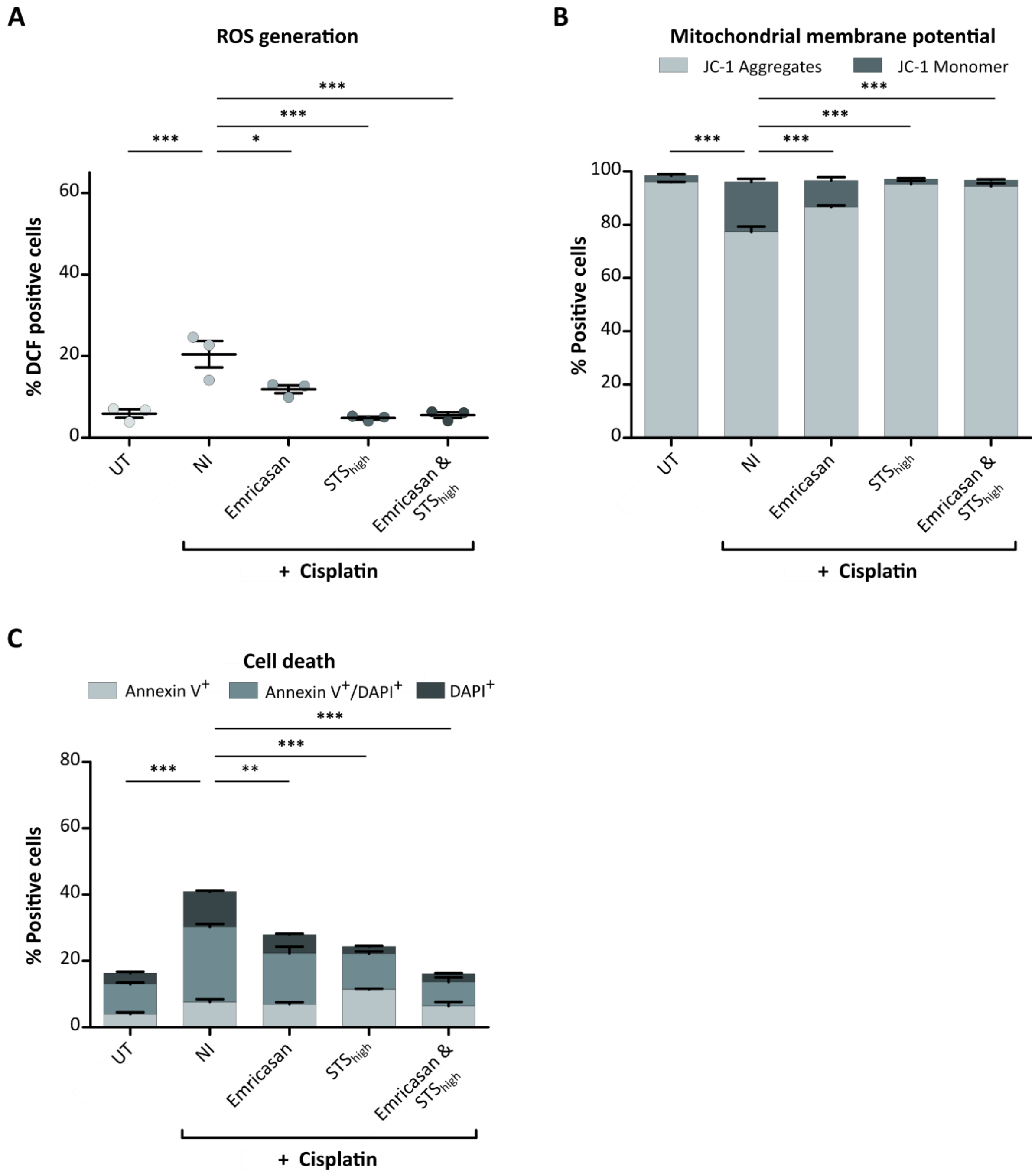

Supplementary Fig. 2 STS_{high} completely prevents cisplatin-induced cytotoxicity in HEI-OC1 cells
A Determination of ROS generation by staining with the non-fluorescent H_2DCFDA (2',7'-dichlorodihydrofluorescein diacetate), which is converted to the highly fluorescent DCF (2',7'-dichlorofluorescein) by cellular esterases and ROS present within the cell. **B** Mitochondrial membrane potential analysis by staining with JC-1, which forms aggregates in healthy cells and remains in a monomeric form upon changes of the mitochondrial membrane potential in unhealthy cells. **C** Analysis of Annexin V^+ (early apoptotic), Annexin $V^+/DAPI^+$ (late apoptotic/dead), and $DAPI^+$ (dead) cells upon cisplatin treatment in HEI-OC1 cells. *N = 3* biological replicates. The data is depicted as mean ± standard deviation (SD) ($P \leq 0.05$ (*), $P \leq 0.01$ (**), and $P \leq 0.001$ (***), determined using one-way ANOVA together with Dunnett's post-hoc test). The performance of cisplatin-treated cells co-cultured with Emricasan, STS_{high} or their combination was statistically compared. Cisplatin treatment: 5 µM, 72 h. Emricasan = 10 µM. STS_{high} = 2 mg/mL. UT = Untreated, NI = No inhibitor.

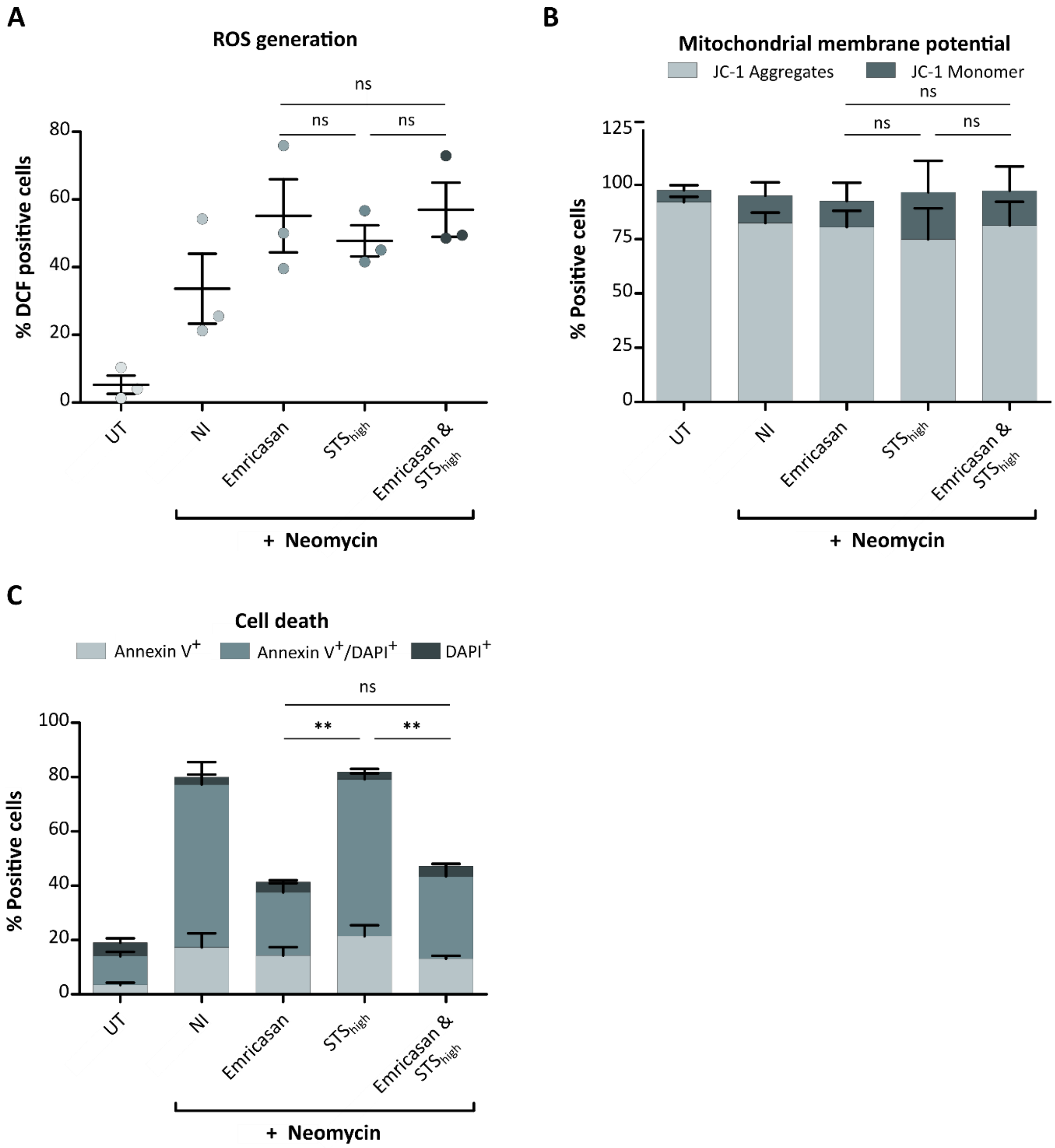

Supplementary Fig 3 STS_{high} does not exert any anti-cytotoxic effect in neomycin-treated HEI-OC1 cells

A Staining of untreated (UT) and neomycin-treated (No inhibitor, NI), Emricasan, STS_{high} and cisplatin co-treated HEI-OC1 cells with the H_2DCFDA probe, which is reduced to DCF in the presence of ROS within the cells. **B** JC-1 staining to distinguish the aggregate (healthy cells) and monomer (unhealthy cells) form of the dye in UT and NI cells and HEI-OC1 cells co-treated with Emricasan and/or STS_{high}. **C** Cell death analysis as determined by staining with Annexin V and DAPI to distinguish early apoptotic (Annexin V^+), late apoptotic/dead (Annexin V^+/$DAPI^+$), and dead ($DAPI^+$) cells. The statistical analysis compared Emricasan and/or STS_{high} co-treated HEI-OC1 cells. N = 3 biological replicates. The data is depicted as mean ± standard deviation (SD) ($P \leq 0.01$ (**), and $P \leq 0.001$ (***), ns = non-significant, determined using one-way ANOVA together with Dunnett's post-hoc test). Neomycin treatment: 2 mM for 72 h. Emricasan = 10 mM, STS_{high} = 2 mg/mL.

Supplementary Material and Methods

Cell culture

Murine HEI-OC1 cells were kindly provided by F. Kalinec (House Ear Institute, Los Angeles, US) and cultured under permissive conditions (33°C and 10% CO_2) in high-glucose Dulbecco's modified Eagle's medium (DMEM; Gibco/Thermo Fisher Scientific, Schwerte, Germany) with 10% heat-inactivated fetal bovine serum (PAN Biotech, Aidenbach, Germany), 100 U/mL penicillin (Sigma Aldrich, Munich, Germany), and 1 mM sodium pyruvate (PAN Biotech). Murine phoenix auditory neuroprogenitors were cultured in ultra-low attachment plates (Corning/Sigma Aldrich) in proliferation medium containing DMEM/F12 (Gibco/Thermo Fisher Scientific) together with 1x N2 and 1x B27 supplement (both Gibco/Thermo Fisher Scientific), 100 U/mL penicillin, 15 mM HEPES (4-(2-hydroxyethyl)-1-piperazineethanesulfonic acid; Pan Biotech), 50 ng/mL heparin sulfate (Iduron, Alderly Edge, UK), human IGF-1 (Insulin-like growth factor 1; 50 ng/mL), human basic FGF (Fibroblast growth factor; 10 ng/mL), and human EGF (Epidermal growth factor; 20 ng/mL) (all Peprotech, Hamburg, Germany). To differentiate the phoenix auditory cells into neurons, cells were cultured in differentiation medium consisting of DMEM/F12, 1x N2 and 1x B27 supplement, 100 U/mL penicillin, 15 mM HEPES, and the cytokine human LIF (Leukemia inhibitory factor; 10 ng/mL), and the neurotrophic factors human NT-3 (Neurotrophin-3; 50 ng/mL), and human/mouse/rat BDNF (Brain-derived neurotrophic factor; 10 ng/mL) (all Peprotech) in Geltrex-coated (Gibco/Thermo Fisher Scientific) cell culture plates. F. Rousset and P. Senn (University of Geneva, Geneva, Switzerland) kindly provided the phoenix auditory cells.

Preparation, cultivation, and identification of neonatal rat primary SGN

In consultation with the German Animal Welfare Act, neonatal Sprague-Dawley rats (P3-5) were used to isolate primary spiral ganglion cells. The registered euthanasia protocol (no.: 2016/118) was performed in accordance with the local authorities (Zentrales Tierlaboratorium, Laboratory Animal Science, Hannover Medical School, including an institutional animal care and use committee) and was reported regularly. The spiral ganglion preparation and the following dissociation using enzymatic and mechanical methods were performed as previously described by Kaiser and colleagues.[1] The isolated spiral ganglion cells were seeded on poly-D/L-ornithine- (0.1 mg/mL; Sigma Aldrich) and laminin- (0.01 mg/mL; Life Technologies, Carlsbad, CA, USA) coated 48-well plates and cultured in serum-free Panserin 401 medium (PAN Biotech) containing 30 U/mL penicillin (Grünenthal GmbH, Aachen, Germany), 6 mg/mL glucose (Braun AG, Melsungen, Germany), 25 mM HEPES (Life Technologies), 3 µg/mL 1x N2 supplement (Life Technologies), and 5 µg/mL insulin (Sigma Aldrich) at 37°C and 5% CO_2. As the spiral ganglion culture not only contains SGNs but also fibroblasts and glial cells, co-staining was performed with Alexa Fluor® 647-anti-CD90.1 (1:200; Biolegend, Koblenz, Germany; #100750) and fluorescein isothiocyanate-anti-nerve growth factor receptor (NGFR) (1:100; Santa Cruz Biotechnology Inc., Heidelberg, Germany; #sc-71691) antibodies for 20 min at 4°C in the dark. In this culture, glial cells express NGFR, whereas fibroblasts express CD90, and we expect SGNs to be negative for both markers. The composition of the spiral ganglion culture with respect to these three cell types was analyzed using the BD FACSCanto™ flow cytometer (BD Biosciences).

Drug treatment

Titrations were accomplished to determine the optimal concentration of the small molecules for further experiments. As a result, drug concentrations used in this study were chosen as follows: 3 µM Muristerone A (R&D Systems, Wiesbaden, Germany; #3816/1), 10 µM Emricasan (Sigma Aldrich; #SML2227), 20 µM Q-VD-OPh (R&D Systems, #OPH001-01M), 20 µM Z-DEVD-FMK (BD Biosciences, Heidelberg, Germany; #550378), 100 nM Ac-DEVD-CHO (BD Biosciences; #556465), and 0.5 µM PFT-α (Tocris Bioscience; #3843/10). The anti-cytotoxic effect of STS (Sigma Aldrich) was tested both using a low (25 µg/mL) and a high (2 mg/mL) concentration. The concentration of cisplatin (Sigma Aldrich, #P4394) was 5 µM for HEI-OC1 cells and phoenix auditory neuroprogenitors and 20 µM for phoenix auditory neurons and primary SGNs. Neomycin (Invivogen, Toulouse, France; #Ant-gn-1) was administered at a concentration of 1 mM to all auditory cells tested if not otherwise indicated.

Cytotoxicity assays

To analyze cytotoxicity upon cisplatin or neomycin treatment and the effects of the selected anti-apoptotic small molecules, different assays investigating cell metabolism or death were performed on HEI-OC1 cells.

To perform the ApoTox-Glo™ Triplex Assay (Promega, Walldorf, Germany) and analyze the dead-cell protease and caspase-3/-7 activity, $2x10^3$ cells were seeded into 96-well Nunc™ MicroWell™ plates (Thermo Fisher Scientific). The next day, the abovementioned concentrations of small molecules and ototoxic drugs were used, and subsequently, the assay was performed 72 h after the addition of the drugs following the manufacturer's instructions. The fluorescence and luminescence were measured using the SpectraMax® Paradigm® Multi-Mode Microplate Reader (Molecular Devices, Munich, Germany).

The Click-iT™ Plus TUNEL (terminal deoxynucleotidyl transferase-dUTP nick end labeling) assay was used to determine DNA fragmentation. The assay uses fluorescently modified dUTP (deoxyuridine triphosphate), incorporated by the terminal deoxynucleotidyl transferase at the 3' end of fragmented DNA. For this purpose, $2x10^4$ cells were seeded on coverslips (Carl Roth, Karlsruhe, Germany) in a 24-well plate (Sarstedt) and treated with the mentioned concentrations of Emricasan and cisplatin. After 72 h incubation, the assay was done according to the manufacturer's instructions. In addition, cell nuclei were stained with DAPI (4’,6-diamidino-2-phenylindole; Sigma Aldrich), and samples were visualized using a phase-contrast fluorescence microscope (Zeiss, Oberkochen, Germany).

To examine ROS generation, cells were seeded at a density of $5x10^4$ cells per well of 12-well plates (Sarstedt) and treated the next day with the indicated concentrations of anti-apoptotic small molecules and ototoxic drugs. The assay was performed with only the still adherent cells in the well with 25 µM of the cell-permeable non-fluorescent H_2DCFDA probe (2',7'-dichlorodihydrofluorescein diacetate; Thermo Fisher Scientific) applied for 10 min at 33°C

and 10% CO_2. After washing and the addition of fresh medium, cells were incubated for 30 min at 33°C and 10% CO_2 to allow reduction of the probe to the still non-fluorescent H_2DCFH by cellular esterases and subsequent conversion to the fluorescent DCF in the presence of ROS. The percentage of DCF-positive cells, indicating the amount of ROS in the sample, was measured using the BD FACSCanto™ flow cytometer.

To analyze mitochondrial health, $5x10^4$ cells were seeded in 12-well plates, and 24 h later, the mentioned concentrations of the small molecules and ototoxic drugs were applied. The analysis was performed 72 h later with the positively charged dye JC-1 (3 µM; MedChemExpress, Sollentuna, Sweden) according to the manufacturer's instructions. In healthy negatively charged mitochondria, the JC-1 dye forms aggregates with red emission at 590 nm, which shifts to a monomeric form with green emission at 529 nm when the mitochondrial membrane potential changes in unhealthy cells. The red-to-green fluorescence ratio was determined using the BD FACSCanto™ flow cytometer.

To analyze the percentage of apoptotic cells in ototoxic drug-treated samples, $5x10^4$ HEI-OC1 cells were seeded in 12-well plates and 24 h later treated with the before-mentioned concentrations of anti-apoptotic small molecules, cisplatin, or neomycin for 72 h. For analysis, dead cells from the medium as well as the still adherent cells were harvested and stained with allophycocyanin (APC)-anti-Annexin V (1:200; BD Biosciences, Heidelberg, Germany, #550474) in 1x Annexin V Binding Buffer (BD Biosciences) for 10 min at room temperature in the dark. After Annexin V staining, cells were additionally stained with 0.2 µg/mL DAPI and immediately analyzed in the BD FACSCanto™ flow cytometer.

Phoenix auditory neuroprogenitors and phoenix auditory neurons were also stained for Annexin V and with DAPI as described above. For this purpose, phoenix auditory neuroprogenitors were seeded at a density of $1x10^5$ cells in ultra-low attachment plates in proliferation medium, and the ototoxic drugs and small molecules were administered directly. After 72 h, the Annexin V assay was performed as described previously. To analyze cell death of phoenix auditory neurons, $5x10^4$ phoenix auditory neuroprogenitors were seeded in differentiation medium on Geltrex-coated plates to allow differentiation for 4 d before the medium was changed, and the small molecules and ototoxic drugs were applied at the concentrations mentioned above. As described above, the proportion of Annexin V^+ cells was analyzed on day 7 of differentiation.

Primary SGNs were cultured in medium containing small molecules and challenged with cisplatin and neomycin the day after culture preparation and seeding of $1x10^4$ cells. FACS staining to distinguish the different cell types present in the culture was conducted 48 h later, as previously described, followed by staining with phycoerythrin-anti-Annexin V (1:200; BD Biosciences; #556422) and DAPI and subsequent flow cytometry analysis.

Live cell imaging

Live cell imaging was used to determine the morphology and confluence of HEI-OC1 or phoenix auditory cell cultures. HEI-OC1 cells were seeded at a density of $5x10^4$ cells per well in 12-well plates, and Emricasan and cisplatin were added the following day. Phoenix auditory neuroprogenitors were seeded at a density of $1x10^5$ cells per well in 24-well ultra-low attachment plates and treated on the same day with Emricasan and cisplatin. Differentiating phoenix auditory neurons were treated with cisplatin and Emricasan on day 4 of differentiation after initially seeding $5x10^4$ phoenix auditory cells in Geltrex-coated wells of 24-well plates. After the small molecule and ototoxic drug administration, live cell imaging was initiated in the Cellcyte X™ device (Cytena, Freiburg, Germany) placed in an incubator (Sanyo, Moriguchi, Japan). The incubator was set to 33°C and 10% CO_2 for HEI-OC1 cells and 37°C and 5% CO_2 for phoenix auditory cells. Images were captured from each condition measured in triplicates every 2 h for 3 d using a 10x (HEI-OC1) or 4x (Phoenix cells) objective in the brightfield analog enhanced contour channel.

Immunoblot

To detect and analyze protein expression or protein phosphorylation status in HEI-OC1 cells, total protein amounts were extracted with a lysis buffer consisting of 50 mM HEPES, 150 mM NaCl (sodium chloride), 50 mM NaF (sodium fluoride), 10 mM $Na_4P_2O_7$ (tetrasodium pyrophosphate), 10% glycerol and 1% Triton X-100. After determining protein concentrations, 30 µg of each total protein sample were loaded onto 10% denaturing polyacrylamide gels for separation by electrophoresis (SDS-PAGE) before blotting the proteins onto a nitrocellulose membrane (GE Healthcare Life Science, Solingen, Germany). Membranes were blocked with 3% milk powder (Carl Roth) in Tris-buffered saline-Tween-20 (TBST) buffer for 1 h at room temperature and then incubated overnight at 4°C with the primary antibodies anti-p53 (1:1000; Cell Signaling; #9282), anti-phospho-p53 (1:1000; Cell Signaling; #9284), or anti-PARP (1:1000; Cell Signaling; #9542) in 3% milk powder in 1× TBST buffer. A goat anti-rabbit IgG antibody coupled to horseradish peroxidase (HRP) was used as the secondary antibody for detection with the SuperSignal™ West Pico Chemiluminescent Substrate (ThermoFisher Scientific). Protein bands were captured using the FUSION FX imaging system (Vilber Lourmat, Eberhardzell, Germany). Membrane staining with HRP-conjugated anti-GAPDH antibody (1:10,000, Biozol, Eching, Germany; #GTX 627408) or anti-vinculin (1:20,000, Sigma-Aldrich, #V9131) followed by HRP-conjugated goat anti-mouse IgG (1:4,000; Cayman Chemical Company, # 10004302) staining confirmed equal protein loading.

Supplementary References

1. Kaiser O, Paasche G, Stöver T, et al (2013) TGF-beta superfamily member activin A acts with BDNF and erythropoietin to improve survival of spiral ganglion neurons in vitro. Neuropharmacology 75:416–425. doi: 10.1016/j.neuropharm.2013.08.008
